# Pulse Granuloma-Like Lesion of the Portal Vein in a Liver Explant: A Case Supporting the Endogenous Theory

**DOI:** 10.7759/cureus.95167

**Published:** 2025-10-22

**Authors:** Eric Young, Keiichi Kinowaki, James P Hamilton, Kiyoko Oshima

**Affiliations:** 1 Pathology, John Hopkins University School of Medicine, Baltimore, USA; 2 Internal Medicine, John Hopkins University School of Medicine, Baltimore, USA

**Keywords:** autoimmune hepatitis with cirrhosis, azathioprine toxicity, liver injury case report, portal vein injury, pulse granuloma

## Abstract

Pulse granuloma (PG) is a rare histopathological finding characterized by eosinophilic hyaline material aggregates and granulomatous inflammation. The pathogenesis of PG remains controversial, with two main theories: the exogenous theory attributing PG to plant-based foreign bodies and the endogenous theory suggesting vascular wall degeneration as the origin. We report a case of PG-like histological features in the portal vein of a patient with autoimmune hepatitis and a history of azathioprine (AZA) treatment, supporting the endogenous theory. The patient was a 49-year-old female with over 10 years of AZA therapy history, who had discontinued AZA treatment four years prior due to cholestatic hepatitis. Histopathological examination of the resected liver revealed portal vein intimal thickening and granulomatous inflammation with eosinophilic hyaline material aggregates and multinucleated giant cells within the intima. These findings resembled PG, but no vegetable matter was identified. This case supports the endogenous theory of PG pathogenesis, suggesting that some lesions traditionally attributed to vegetable matter may instead result from vascular damage and subsequent collagen degeneration.

## Introduction

Azathioprine (AZA) is an immunosuppressive agent widely used in the treatment of autoimmune diseases. However, AZA is known to cause chronic liver injury, including sinusoidal obstruction syndrome and nodular regenerative hyperplasia [1]. These hepatic injuries are primarily attributed to AZA-induced vascular endothelial cell injury.

Pulse granuloma (PG) is a rare histological lesion that commonly occurs in the gastrointestinal tract, oral cavity, and lungs [2]. The pathogenesis of PG remains controversial, with two competing theories: the "exogenous theory" and the "endogenous theory" [3]. The exogenous theory posits that PG develops as a tissue reaction to plant-based foreign bodies, such as legumes, and is often associated with conditions like gastrointestinal perforation and aspiration pneumonia [4]. This theory is supported by cases where plant material has been identified within the lesions. Conversely, the endogenous theory suggests that PG originates from hyaline degeneration of blood vessel walls, leading to the formation of eosinophilic hyaline material that elicits a granulomatous response [3,5]. Histologically, PG is characterized by aggregates of eosinophilic hyaline material and inflammatory cell infiltration with multinucleated giant cells [6].

DeRoche et al. reported five cases of PG in the gastrointestinal tract and gallbladder, supporting the exogenous theory as plant material was identified in all cases [4]. However, PG has also been reported in sites where the presence of plant material is unlikely, such as the gallbladder, fallopian tubes, skin, and prostate [3,7]. Pilatti et al. have suggested that vascular changes may lead to the formation of PG-like histological features, with an example of perianal PG [5].

We report a case of portal vein injury with histological features resembling PG in a patient who received long-term AZA treatment. This case provides evidence supporting the endogenous theory of PG pathogenesis, as no plant material was identified, and the lesions were associated with vascular injury.

## Case presentation

The patient was a 49-year-old female who had been treated with AZA for autoimmune hepatitis for over 10 years. Four years prior, she developed cholestatic hepatitis, leading to the discontinuation of AZA treatment. Subsequently, she was treated with mycophenolate mofetil and ursodeoxycholic acid but developed refractory ascites. Six months before presentation, she underwent transjugular intrahepatic portosystemic shunt placement from the right portal vein branch of Segment 8 to the right hepatic vein. The patient had no history of major gastrointestinal surgery or perforating gastrointestinal injury. She ultimately received a living donor liver transplantation.

**Figure 1:**
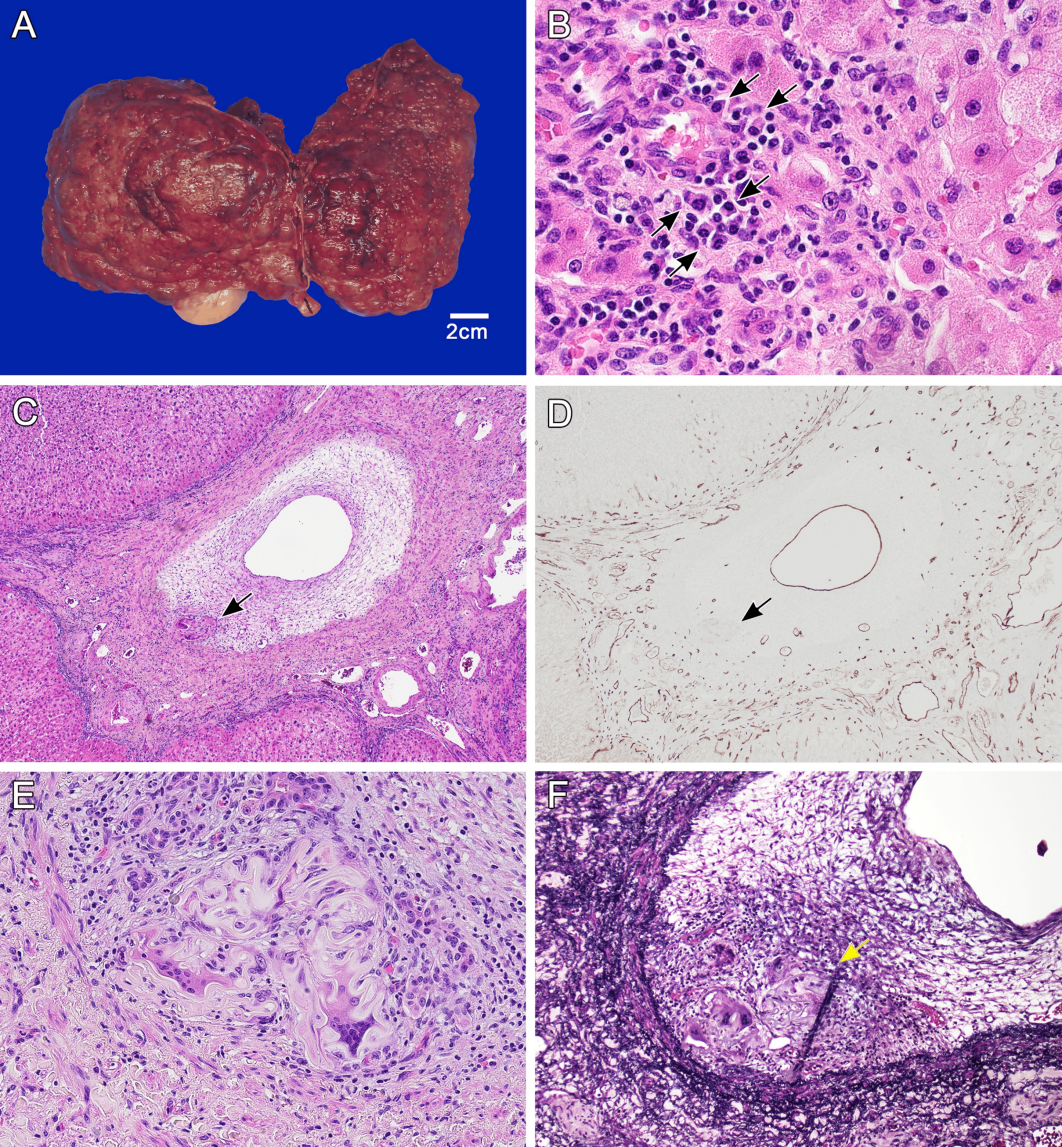
Gross and histologic features of liver explant from patient with autoimmune hepatitis treated with AZA Gross and histologic features of liver explant from patient with autoimmune hepatitis treated with AZA. Gross appearance of cirrhotic liver (A, B). Hematoxylin and eosin staining demonstrating portal vein stenosis and nodular aggregates of ribbon-like eosinophilic hyaline material within intimal sclerosis (arrow) (C). Anti-CD34 immunostaining highlights endothelial cells of the portal vein and small vessels within intimal sclerosis (D). High-magnification image shows a granulomatous inflammatory reaction with multinucleated giant cells and mononuclear cell infiltration surrounding the hyaline material (E). Movat pentachrome stain highlights the internal elastic lamina and confirms the location of the nodule within intimal sclerosis (F). AZA, azathioprine

Macroscopically, the explanted liver exhibited features of macronodular cirrhosis, with multiple regenerative nodules visible on the cut surface (Figure 1A). Histopathological examination confirmed established cirrhosis. The portal tracts and lobules showed mild chronic inflammation with increased plasma cell infiltration, consistent with autoimmune hepatitis (Figure 1B). The two foci of the medium-sized portal veins demonstrated marked fibrous intimal thickening. Within the thickened intima, H&E staining revealed nodular aggregates of ribbon-like eosinophilic hyaline material in Segment 5 (Figure 1C and Figure 1E). Surrounding this hyaline material was a granulomatous inflammatory reaction with multinucleated giant cells and mononuclear cell infiltration. These findings closely resembled the characteristic features of typical PG. CD34 immunohistochemistry was performed to highlight endothelial cells, and Movat pentachrome staining, which renders elastic fibers black, was used to visualize vascular structures and localize the nodular lesion within intimal sclerosis (Figure 1D and Figure 1F). No foreign material was observed, including plant-derived substances exhibiting the characteristic lamellar or helical structures of plant cell walls or suture-related materials, with confirmation also performed using polarized light. Furthermore, no similar lesions were observed in the hepatic artery or the bile ducts at the hilar region and central veins.

## Discussion

PG is a rare condition diagnosed by its characteristic histopathological features. According to DeRoche et al., the typical histological appearance of PG includes eosinophilic hyaline rings and granulomatous inflammation centered around multinucleated giant cells, often accompanied by plant material [4]. The lesions observed in the portal vein of our case exhibited aggregates of eosinophilic hyaline material and multinucleated giant cell reactions, closely resembling typical PG. No foreign bodies were identified, distinguishing this case from previously reported intrahepatic portal vein foreign body granulomas [8].

The pathogenesis of PG remains controversial, with two main theories: the "exogenous theory" and the "endogenous theory" [3]. The exogenous theory posits that plant material, such as legumes (pulses), enters the tissue and elicits a foreign body reaction [3,4]. Specifically, the cellulose component of plant cell walls, which is indigestible in humans, can persist in tissues for extended periods, leading to granuloma formation [3]. Talacko et al. successfully reproduced lesions similar to human PG in rats by injecting legume homogenate into the oral submucosa [9]. Conversely, the endogenous theory suggests that hyaline degeneration of blood vessel walls can give rise to PG-like lesions [3,5]. This theory is supported by cases where PG-like lesions occur in locations where plant material exposure is unlikely.

AZA is a prodrug of 6-mercaptopurine, metabolized in vivo by glutathione S-transferase [1]. Widely used as an immunosuppressant, AZA is known to cause vascular endothelial cell injury by enhancing oxidative stress through glutathione depletion during its metabolism [10]. Indeed, portal flow disorders such as sinusoidal obstruction syndrome and nodular regenerative hyperplasia have been reported in patients receiving AZA [1]. In our case, the patient had a history of long-term AZA administration, which was discontinued due to cholestatic hepatitis, clinically consistent with AZA-associated liver injury. The PG-like lesions in the portal vein may have resulted from vascular endothelial cell injury, including AZA-induced injury, followed by degeneration of vascular wall collagen. In addition, six months before liver transplantation, a transjugular intrahepatic portosystemic shunt was performed. The shunt was created from the right portal vein branch of Segment 8 to the right hepatic vein, whereas the PG was identified in Segment 5. Because these sites are anatomically distant, a direct connection is unlikely. However, the possibility that the shunt formation contributed to the lesion cannot be ruled out. In this case, no plant material was identified, and the lesion was clearly localized within the vascular wall. Although it is difficult to determine whether the lesions were primarily due to vascular endothelial injury, including that induced by AZA, or to the effects of shunt formation, both possibilities support an endogenous rather than an exogenous origin of the portal vein thrombus-like lesions. While the "exogenous theory" of PG is currently predominant, our case demonstrates that vascular wall injury can lead to the development of PG-like lesions. As Philipsen et al. noted in their review, the pathogenesis of PG may not be singular, but potentially involves both exogenous and endogenous factors [3]. Notably, our case suggests that some PGs traditionally attributed to plant-based foreign bodies may actually be based on vascular injury.

The temporal relationship between AZA discontinuation and the appearance of PG-like lesions in our case is noteworthy, as the interval was four years. AZA-induced vascular injury is generally regarded as a chronic, cumulative process rather than an acute insult. Experimental studies have demonstrated that granuloma formation progresses gradually, from initial cellular injury to mature granulomatous lesions over weeks to months [9]. In our patient, more than a decade of AZA exposure likely resulted in sustained endothelial and vascular wall damage that persisted even after cessation. The metabolite 6-mercaptopurine induces oxidative stress and glutathione depletion, thereby creating a long-lasting pathological environment conducive to vascular wall degeneration [1]. Consequently, the four-year interval may represent the natural course of progressive collagen degradation and hyaline transformation within the vascular wall. The reported timeframe for AZA-induced endothelial injury is variable: acute manifestations such as sinusoidal obstruction syndrome and hepatotoxicity may develop within weeks to months of treatment initiation [10], whereas chronic complications such as nodular regenerative hyperplasia and portal hypertension typically arise after years of exposure [1]. The incidence of AZA-related hepatotoxicity in autoimmune hepatitis is considered uncommon, with the more severe vascular complications even more rarely documented. To our knowledge, PG-like vascular lesions have not previously been described in the context of AZA therapy, suggesting an exceptionally rare manifestation requiring multiple converging factors.

Recently, Gonzalez et al. investigated the incidence and characteristics of PG in the small and large intestines, reporting that some PGs develop without obvious plant material involvement [6]. Additionally, Rhee et al. reported extraintestinal PGs in the gallbladder, fallopian tubes, and skin, demonstrating that PG can occur without direct exposure to plant material [7]. These findings further support the endogenous theory and suggest that PG pathogenesis may be more complex than initially thought.

This case presents a novel histopathological manifestation supporting the endogenous theory of PG pathogenesis and offers a new perspective on understanding PG development. Additional investigations are needed to elucidate the relative contributions of exogenous and endogenous factors in PG pathogenesis.

## Conclusions

This case highlights a rare presentation of PG-like lesions within the portal vein in a patient with long-term AZA treatment for autoimmune hepatitis. The absence of plant-derived material and the clear localization of the lesions within the vascular wall support the endogenous theory of PG pathogenesis. These findings suggest that vascular injury, particularly that induced by chronic AZA exposure, may play a critical role in the development of PG-like histological features. Our report contributes to the growing body of evidence indicating that PG may arise through mechanisms independent of exogenous plant material, underscoring the need for a broader understanding of its pathogenesis. Further studies are warranted to clarify the relative contributions of endogenous vascular injury and exogenous factors in the formation of PG.
